# Evaluation of Children’s Thermal Environment in Nursery School: Through the Questionnaire and Measurement of Wearable Sensors Approach

**DOI:** 10.3390/ijerph19052866

**Published:** 2022-03-01

**Authors:** Xin Yuan, Yuji Ryu

**Affiliations:** Faculty of Environmental Engineering, The University of Kitakyushu, Hibikino1-1, Wakamatsu-ku, Kitakyushu 808-0135, Japan; uanin03@gmail.com

**Keywords:** children, indoor thermal environment, wearable sensor, adaptive comfort behavior

## Abstract

Due to psychological and physical differences, children are more vulnerable to the influence of the surrounding environment than adults. A nursery school in Japan was selected as the research object. The actual thermal environment of children aged 1 to 5 in the classroom was evaluated based on measured data in winter and summer. Through a questionnaire survey of nursery teachers, this paper analyzed and compared the relationship between teachers’ thermal adaptation behavior and children’s thermal sensation. Compared with the traditional fixed-points measurement method, a method of wearable sensors for children was proposed to measure the indoor temperature distribution. The traditional measurement results showed that 73% of classroom indoor temperatures and humidity do not meet the thermal comfort standard stipulated by the government. The method proposed in this paper indicates that: (1) nursery teachers’ thermal adaptation behavior may not be based on children’s thermal sensations; (2) solar radiation and weather context could lead to uneven indoor horizontal temperature distribution, hence, specific attention should be paid to the thermal environment when children move to the window side; and (3) the density of occupants causes the temperature around the human body to be relatively high. We suggest that teachers improve the thermal comfort of gathered children through thermal adaptive behaviors. The results of the study provide valuable information for nursery managers to formulate effective indoor thermal environment strategies from the perspective of children.

## 1. Introduction

The indoor environment has a great impact on indoor occupants’ physical and mental health and work efficiency [1,2]. At present, most standards and research are focused on creating an appropriate indoor environment for adults. Meanwhile, due to the underdeveloped thermoregulatory function of children and other problems, they are more vulnerable to the impact of the environment on health than adults. Thus, children are considered a vulnerable group [3]. Recently, research on children has become an important issue [4,5,6,7,8]. Nursery schools are the main places for children to study, play, socialize, and other functions. Therefore, whether a nursery school can create and maintain a comfortable indoor environment for children had attracted more and more attention [9,10,11].

Current studies had investigated the implications of the indoor environment of nursery schools on children from various aspects. For example, Madureira et al. [9] found that the quality of the indoor environment has a strong influence on health and learning through people’s perception and satisfaction. Branco et al. [1] investigated the correlation between air pollution and childhood asthma and concluded that the indoor thermal environment is the most important parameter in indoor air quality. An optimal indoor thermal environment could contribute to reducing the risk of overheating and provide suitable indoor thermal conditions [2], which could help with children’s health and learning [10,12]. However, as most research focused on studying the indoor environment and children’s thermal comfort in naturally ventilated rooms [13,14,15,16], there is still a lack of research on children’s thermal comfort in air-conditioned and mechanically ventilated rooms. It is worth noting that there are differences in thermal feeling and acceptance between users in naturally ventilated rooms and air-conditioned rooms. In air-conditioned rooms, occupants usually have higher requirements for indoor thermal conditions [17]. Hence, in this work, we focused on the thermal environment for children in air-conditioned rooms. 

So far, research on how to improve children’s thermal environment has mostly been based on in-site measurements or questionnaire surveys [18]. In studies based on field measurements, the measurements were mostly conducted in or at several fixed points in the classroom [14,19]. However, the classroom layout could result in non-uniform thermal zones due to solar radiation, diverse thermal radiant fields caused by cold/hot surfaces, and draughts. Meanwhile, the sparsely distributed points of measurement could not represent the temperature distribution in a room. Therefore, local discomfort evaluations in relation to subjects’ position in the room are necessary [18]. In addition, infrared thermal sensors and images can be used to identify individual thermal environments [20], through monitoring physiological signals, such as the temperature of the skin or face, on certain parts of the body [21]. For example, Cosma et al. [22,23] measured local body temperature with thermal images and modeled the individual thermal comfort under transient conditions. Wang et al. [24] used the online learning thermal comfort model of infrared thermal images to control the thermal environment. Although the thermal sensor and camera can be installed or set on the wall or the ceiling, there are some limitations, such as that the sensor or camera must be placed in front of the subject within a fixed, relatively small distance. This indicates the inapplicability of thermal images if the subjects could have various activities and locations in the actual operating environment [25]. Moreover, Sugimoto [26] proposed a wearable system for measuring biological data, activity data, and location data in human daily life. The wearable sensors have recently been used to monitor physiological signals, because of the advantages of small interference with the measured object and no need for any cooperation with the measured object [25,27]. Therefore, to capture the real situation of children, we used micro wearable sensors to measure the surrounding environment of children and evaluated the corresponding thermal environment. Simultaneously, we used a thermography camera to record the thermal conditions within each classroom.

In addition to field measurements, subjective investigation is another important method to investigate the thermal environment in nursery classrooms [18]. Yun et al. [11] studied the influence of variables on the difference in children’s thermal comfort through a questionnaire survey targeting 119 nursery school children aged from four to six. Fabbri [12] studied children’s subjective judgment according to the questionnaire modified by the method of psychological education. Korsavi et al. [6] conducted a questionnaire survey and observation table for 805 children in primary schools to record their thermal comfort and adaptive behaviors. They concluded, during the heating season, that the comfortable temperature for children is relatively lower than expected, and children are more sensitive to temperature changes. This can be attributed to children’s lower personal behavior practices and more consistent indoor conditions during the heating season. During the heating season, the proportion of children engaged in personal behavior is one-third lower. Among them, about 80% of window operations are carried out by teachers, who have a higher comfortable temperature than children. In other words, students do not have autonomy in terms of thermal comfort and these conditions may lead to their low thermal comfort. Meanwhile, children have a slower rate of heat acclimation compared to adults and are at higher risk of developing heat illness when preventive measures are not taken in a timely manner [28]. Berko et al. found that children are susceptible to heat stress, with increased morbidity or mortality compared to a healthy adult reference population [29]. In addition, children’s skin temperature was lower in cold environments, reflecting greater vasoconstriction. Their metabolic heat increases more in cold environments than in adults, which leads to a situation where children may not be able to maintain their body temperature sufficiently during prolonged rest, which would directly affect the thermal comfort of children [28,30]. The reasons contributing to this situation are mainly due to the immaturity of children’s physiological systems, morphological, and neuroendocrine as well as the marked differences in adult-child thermoregulatory responses to environments [28,31]. It indicates that the current adult-based comfort standards should not be applicable to children [32,33]. Therefore, to provide a suitable indoor environment for children, especially for children aged 1 to 5 who lack thermal adaptive behaviors, it is more important for nursing teachers to evaluate indoor thermal comfort and recognize children’s thermal comfort. Hence, a questionnaire survey was conducted among teachers in nursing work to capture the thermal comfort and adaptive behavior of the teachers and whether the thermal adaptive behaviors are carried out based on children’s thermal comfort.

In this paper, we conducted a questionnaire survey on nursery teachers at a total of 183 (recovery rate was 38.8%) nurseries in Fukuoka, Japan. Based on the statistics of the questionnaire, we selected a common case for the actual measurement survey. Specifically, the indoor temperature was measured at four fixed points at the heights 0.1 m, 0.3 m, 1.1 m, and 1.6 m above the ground in each classroom, and tiny wearable sensors were put into children’s right thigh pants pockets and the temperature in the pocket was measured to represent the actual temperature around the child. The thermal environment of children in summer and winter was assessed by measuring the indoor temperature at a fixed point and the actual temperature around the children and observing their behavior and position in the classroom. The objectives are (1) to investigate whether teachers’ thermal comfort and thermal adaptation behavior are child-centered, (2) to explore the thermal environment of children in air-conditioned rooms and discuss the differences from fixed points of indoor air temperature measurements, (3) to capture the actual surrounding temperature for each child both in summer and winter with wearable sensors, and (4) to evaluate the thermal environment from the perspective of children. The study results emphasize the importance of creating a suitable indoor environment strategy from the practical perspective of children’s thermal environment while providing valuable information for designers and nursery managers to formulate effective indoor thermal environment strategies from the perspective of children.

## 2. Methods

The research method is summarized in two aspects: In the first phase, we conducted a questionnaire survey of teachers working in nursing care in a total of 183 (recovery rate was 38.8%) nursery schools in Fukuoka, Japan. We specifically focused on teachers’ thermal comforts and adaptive behaviors in nursing care and investigated whether teachers’ thermal adaptive behaviors were child-centered.

**Figure 1:**
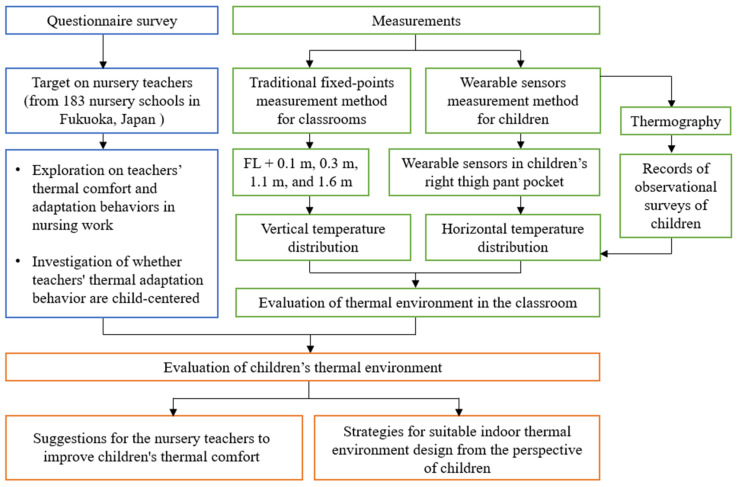
Flow chart of the study’s methodology. Source: Own source.

In the second phase, we selected a comment nursery school for the study based on the statistical results of the questionnaire survey. Through a combination of traditional fixed-point measurements of classrooms and measurements of children wearing wearable sensors, we conducted field measurements of classrooms and children from one year old to five years old in nursery school in winter and summer. The flow of this study was shown in Figure 1, and the content will be further detailed in Section 2.1 and Section 2.2.

### 2.1. Questionnaire Survey

Taking 183 facilities with more than 100 people in licensed nursery schools in Fukuoka City as the objects, a questionnaire survey was conducted on the teachers who take care of children. The questionnaire was mailed on 11 October 2019, and 25 October 2019 was the response date. The objectives of the questionnaire survey were to explore teachers’ thermal comfort and adaptation behavior in nursing work and to investigate whether the thermal adaptation behavior is child-centered. The questionnaire consists of a total of 25 questions and the questions include two aspects: (a) about the nursery school (with/without shading equipment and the type of shading equipment, the type/set temperature of the air conditioning system, ventilation mode); and (b) about the indoor thermal environment (satisfaction with the thermal environment, vertical/horizontal temperature distribution, problems in indoor thermal environment, and improvement methods when using air conditioning). The questionnaire recovery rate was 38.8% (71/183).

### 2.2. Measurements

#### 2.2.1. Case Study of a Nursery School

The case study nursery school is in Fukuoka city, Japan. Fukuoka city, which is at 130.42° N, 33.58° E, is one of the largest cities in Japan. The average annual temperature of the city is about 16 °C, peaks in August at about 27–28 °C, and reaches its minimum in January at about 5–6 °C. Meanwhile, the city has a humid climate throughout the year with the annual precipitation ranging from 1600 to 2000 mm (www.jma.go.jp (accessed on 10 December 2021)). Table 1 shows the weather during the field study in August and September 2019 and February 2020.

This nursery school is the target facility of NTT (Japan telegraph and telephone west corporation) West Japan, Fukuoka City working to realize IoT (Internet of things) nursery school [34]. The business of nursery schoolteachers involves many aspects. By carrying out the business of using IoT/IT technology and adding IoT to the air conditioner, air visualization and automatic control can be realized, thereby reducing the business of nursery schoolteachers. The information of the object nursery school is shown in Table 2.

#### 2.2.2. Measurement

**Figure 2:**
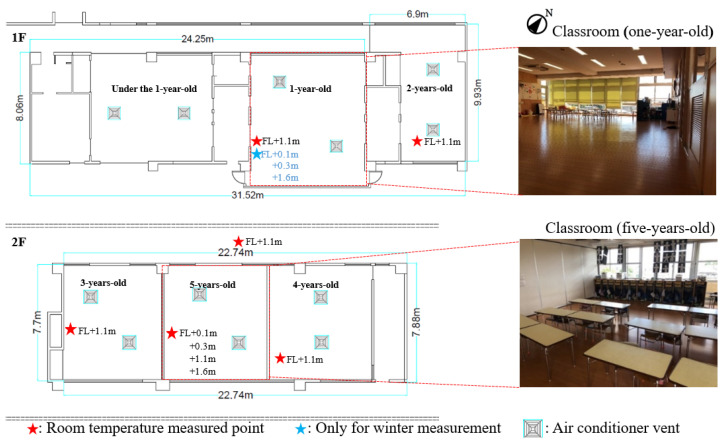
Profile of measurement sites. Source: The figure is taken and made by authors.

**Figure 3:**
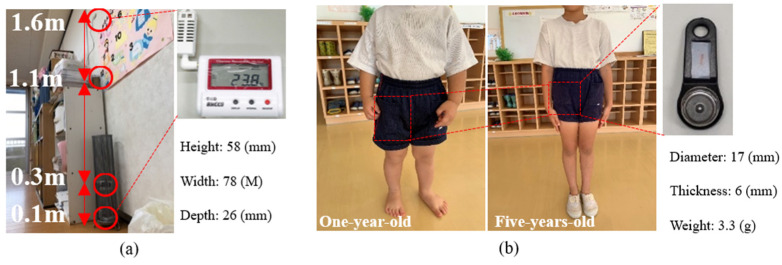
Profile of measurement instruments. (**a**) Measured points and data logger; (**b**) clothing for children, and the mini-size wearable sensor. Source: The figure is taken and made by authors. The parameters for the data loggers are from T&D Corporation (https://www.tandd.co.jp/product/spec/outline-spec-tr7wb-nw-jpn.pdf (accessed on 25 February 2022)) and the mini-size wearable sensors are from KN Laboratories, Inc. (https://www.kn-labs.com/hygrochron.htm (accessed on 25 February 2022)).

The measurements were conducted from 28 August to 9 September 2019, and from 19 to 25 February 2020. Temperature and humidity data loggers were set in classrooms for children aged from 1 to 5 and used to record the indoor air temperature and humidity of each classroom. Meanwhile, the temperature and humidity data loggers were also set on the outdoor balcony to record the outdoor air temperature and humidity. Since the children may touch or damage the data recorders, and to minimize the interference with the children’s class, we put the data recorders on the side near the middle of the wall in each classroom (Figure 2). At the same time, the temperatures at four heights above the ground in the classrooms of one-year-old and five-years-old children, i.e., 0.1 m, 0.3 m, 1.1 m, and 1.6 m, were recorded (Figure 2 and Figure 3). In addition, to better understand the actual temperature around the children, we put mini-size wearable sensors (17 mm diameter and 3.3 g weight) in the right thigh pants pockets of children aged one and five to measure the temperature around the children (Figure 3b). The profiles of the instrument parameters for the measurements are shown in Table 2. In addition, we also observed and recorded the behaviors and positions of the children with the wearable sensors. Meanwhile, we used a thermography camera to record thermal conditions in the classrooms, and the parameters of the camera are shown in Table 2. In this study, since the temperature in the pocket of the right thigh of the pants was measured, to eliminate the error caused by clothing, the clothing types were unified both in summer and winter (Figure 3b). During measurements, one-year-old children were wearing diapers and shorts, and five-years-old children were wearing underwear and shorts. The pants were all made of the same material, while the individual diapers and underwear were different.

## 3. Result and Discussion

### 3.1. Result of Questionnaire Survey

As shown in Table 3, teachers responsible for children under one year old answered the questionnaire the most. In addition, some nursery schools do not set up separate classrooms or separate teachers for children aged from 0 to 3. The results showed that 87% of the air-conditioning systems used in the facilities are the ceiling cassette type, which could be owing to the installation of the air-conditioning system being considered at the design stage of the nursery school, as the installation of air-conditioning system is essential for children to spend their time comfortably. In addition, 59% of the facilities use floor heating, and most of the facilities use ceiling cassette and floor heating in winter. The result showed a great difference in the indoor thermal comfort and satisfaction when air conditioning is operating or not. Most respondents feel comfortable and satisfied when air conditioning is operating and feel uncomfortable and dissatisfied when air conditioning is not operating.

In general, the thermal environment of using an air conditioning system makes indoor occupants feel more comfortable. Hence, the air conditioning system could have a great influence on the creation of a comfortable indoor thermal environment. Due to the operation, mode, and temperature setting mostly being based on the thermal feelings of the teachers, 20.9% of respondents have the question about whether the set temperature matches the temperature of the child’s space, and 17.6% of respondents worry about whether the children feel comfortable or not. We highlight the importance of improving the teachers’ cognition of children’s thermal comfort. Moreover, more than half of the childcare workers feel the vertical and horizontal temperature difference indoors. In particular, 89% of facilities feel the horizontal temperature difference between the parts exposed to the sun and the parts not exposed to the sun (Table 4). In addition, even though 94% of nurseries were equipped with sunshades, temperature differences were still perceived indoors. As Table 3 shows, there were also cases of changing the operation and temperature adjustment of the air conditioning system based on the judgment of children’s physical conditions. However, the most common answer was to adjust the air conditioning system according to the teacher’s feeling. Therefore, nursery schoolteachers had the problem of not knowing whether the set temperature of the air conditioner matches the temperature of the child’s space. Therefore, it can be inferred that it may be inappropriate to set and adjust the temperature of the air conditioner according to the teacher’s own feeling. It is important to set the appropriate temperature of the air conditioning system based on creating a comfortable space for children.

### 3.2. Results of Measurements

The measurements were conducted during school days from 28 August to 9 September 2019 in summer, and from 17 to 24 February 2020, in winter. Due to the different times of children entering and leaving the nursery school every day, the measurement started at 9:00–10:00 and ended at 15:00–16:00. Table 5 shows the indoor temperature adjusting methods during the field measurement period. Except for 19 February 2020, floor heating and air conditioning were used for heating in the one-year-old children’s classroom, and the indoor temperature was adjusted by air conditioning on the other days. According to the Japanese Ministry of Health, Labour, and Welfare [35], the thermal comfort zone of a nursery school classroom is 26–28 °C in summer and 20–23 °C in winter, and according to the regulations of the Ministry of Health, Labour, and Welfare (Japan) [36], the standard indoor humidity is 40–70%. This paper adopts this standard to evaluate the thermal environment in the classroom.

**Figure 4:**
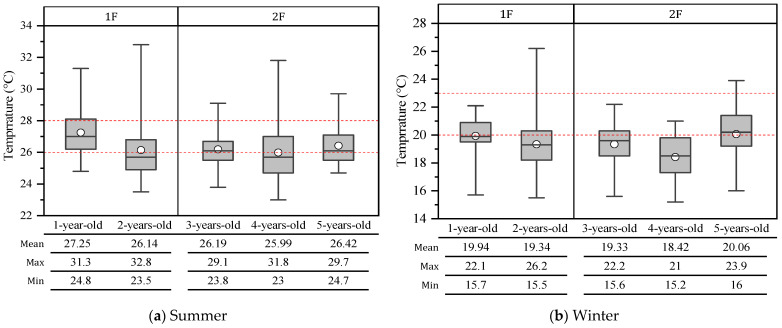
Indoor temperature of children’s classroom at the FL (Floor) + 1.1 m in classrooms for children aged from 1 to 5. (**a**) In summer; (**b**) in winter. Source: Own source.

Due to the height of the tallest child being 1.1 m, 1.1 m from the floor was selected as the height to measure the indoor temperature. Figure 4 shows the indoor temperature of the children’s classrooms during summer and winter measurements. Except for the classroom for 1-year-old children, indoor temperatures in summer were often distributed outside of the thermal comfort range designated by the Japanese government. The frequency of the temperature of the classroom for one-year-old children meeting the thermal comfort in summer was the highest, which may be due to the layout of the nursery, directing sunlight through the whole window on the west wall. Meanwhile, the results showed that children were more frequently staying in an uncomfortable thermal environment, especially in winter, and the dissatisfaction rate of the children reached 60%. It indicated that the children could have a strong sense of coldness in the classroom. In addition, children have the physiological characteristic of being easier to cool in a cold environment [31], and indoor temperature over-cooling deteriorates thermal comfort [37]. Hence, in this situation, the actual thermal comfort of children might be lower than expected.

**Figure 5:**
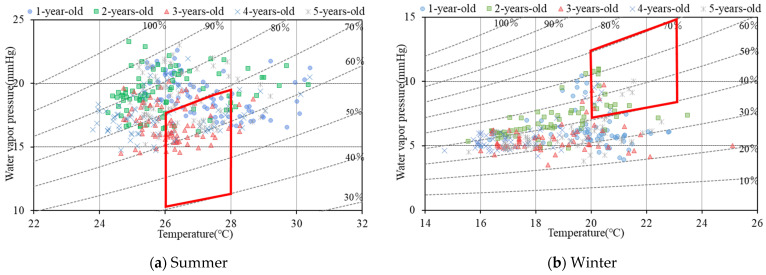
Indoor air temperature and humidity distribution every 2 h in the classroom for children aged 1 to 5 at FL + 1.1 m. (**a**) In summer; (**b**) in winter. Source: Own source.

The classroom was prone to low temperature and high humidity in summer and low temperature and low humidity in winter (Figure 5). The dissatisfaction rate of the temperature and humidity reached 63% in summer and 86% in winter. In addition, studies over the past few decades have shown that there is a direct relationship between indoor humidity and occupant health. Too low or too high indoor humidity may lead to physical discomfort because the relative humidity directly affects the perception of comfort [38]. Common sanitary indicators of high humidity include visible mold, wet stains, condensation on walls and windows, odor, and smells [39,40,41]. The health effects of low humidity include pathogens and disease transmission [42,43] and are also related to nasal airway and laryngeal airway dryness, hand dryness, and eye irritation [43,44,45,46]. In addition, low relative humidity can lead to high fatigue, reading speed, and distraction [47]. Therefore, improving indoor temperature and humidity is important for the nursery to create and maintain a comfortable indoor thermal environment. The indoor temperature and humidity can be adjusted through comfort adaptive behaviors such as using ventilation or set dehumidifiers in summer, and a humidifier in winter.

This section analyzes the current situation of indoor thermal environments through the traditional fixed-point measurement in the classroom for children aged one to five. Through the statistical analysis, the results showed that the thermal environment of the classrooms could not meet the standard across 73% of school hours. However, due to the indoor temperature distribution being more likely to be uneven, the measurement was conducted only at a fixed point, which is not universal and cannot represent the indoor temperature distribution. Therefore, this work optimized the non-universality of the current measurement methods. In this work, we selected the 1-year-old and 5-year-old children with the largest age difference in the nursery as the objects. Meanwhile, the indoor temperature in summer and winter was measured by four fixed points at different heights from the ground, and the wearable sensors worn by children were used to evaluate the real thermal environment around the children in the classrooms.

#### 3.2.1. Distribution of Vertical Temperature

**Figure 6:**
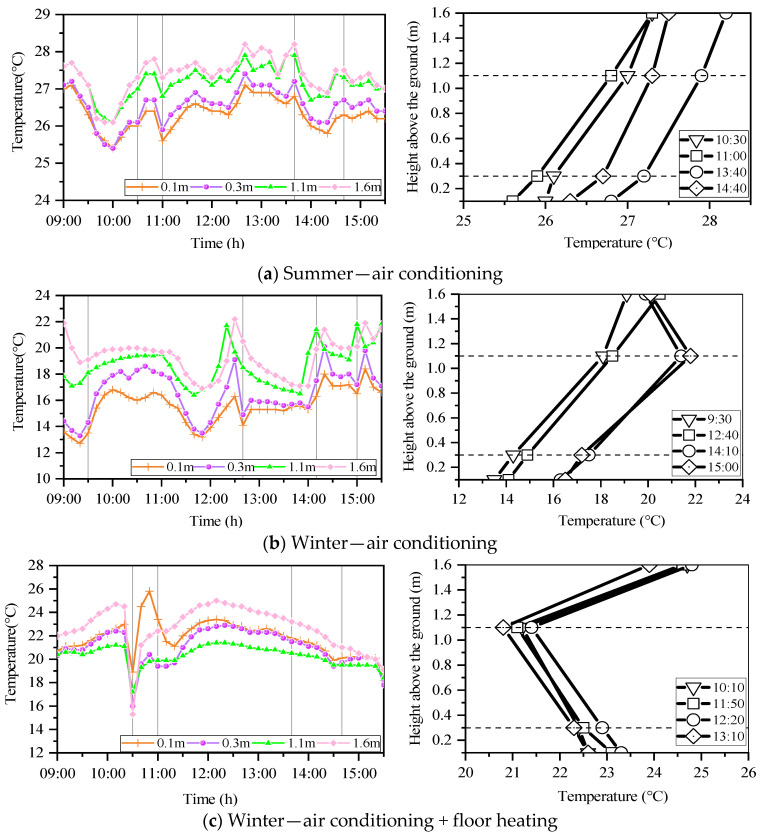
Typical diurnal indoor air temperature at different height variations. (**a**) In 9 September 2019; (**b**) in 19 February 2020; (**c**) in 19 February 2020. Source: Own source.

Figure 6 shows the results of the indoor vertical temperature distribution. When the indoor temperature was adjusted by the air conditioning only, the vertical temperature distribution showed that the indoor air temperature was high near the ceiling and low near the floor. As Figure 6a (left side) shows, after adjustment, the temperature drops to about 25.5 °C sharply at 10:00. At 11:00, the temperature difference between the upper and lower heights reached its highest, with a difference of 1.8 °C, which was caused by the cooling operation.

Temperature fluctuation was observed in Figure 6b (left side), which could be caused by the children’s schedules of going to the other classrooms and outdoor activities. Thus, the indoor temperature could be greatly affected by the outdoor temperature when children are entering or leaving the classroom. Except that the air conditioner is turned on at 9:00, there may be an abnormal temperature, which is not within the analysis range. The vertical temperature difference can be observed all day. Meanwhile, in Figure 6b (right side), the four time-points with the largest vertical temperature differences between 0.1 m and 1.1 m were selected and analyzed. The figure shows that the temperature difference between 0.1 m and 1.1 m (the height of the child’s line of sight [48]) reached a maximum of 6.2 °C. According to ISO 7730 [49], the allowable vertical temperature difference is 3 °C, while the non-compliance rate for the vertical temperature difference shown in this classroom is 50%. This result shows that half the time, children are in an environment with different temperatures around their feet and around their heads, which is not a comfortable thermal environment for children.

In addition, both floor heating and air conditioning were used for heating in the classroom (Figure 6c (left side)), and the room temperature at a 1.1 m height over the floor was the lowest, while that at 0.1 m and 0.3 m over the floor were relatively high, which results in a different vertical temperature distribution than the other days. At the same time, except for the sharp drop in room temperature caused by opening windows for ventilation around 10:30, the results on the right side of Figure 6c show that the indoor vertical temperature difference is within the standard of ISO 7730.

Therefore, measurements of the classrooms indicated that there is a vertical temperature distribution in the classroom. Additionally, it shows that the simultaneous use of floor heating and air conditioning could significantly reduce the indoor vertical temperature distribution and help the nursery create and maintain a comfortable thermal environment for children.

#### 3.2.2. Distribution of Horizontal Temperature

**Figure 7:**
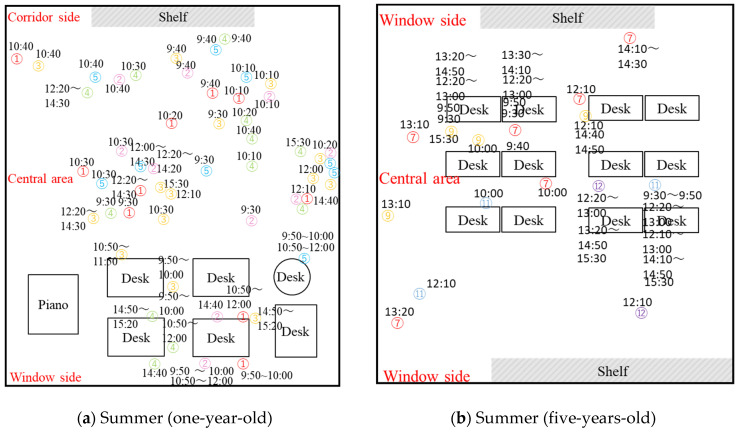

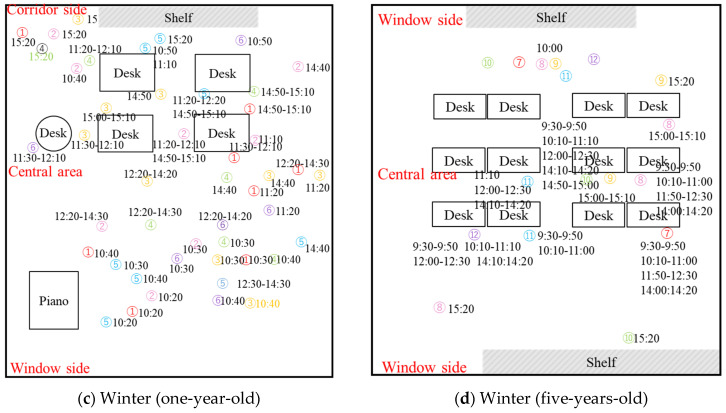
Records of typical observational surveys of children. (**a**) Records of typical summer observational survey of one-year-old; (**b**) records of typical summer observational survey of five-years-old; (**c**) records of typical winter observational survey of one-year-old; (**d**) records of typical winter observational survey of five-years-old. Source: Own source.

To investigate the temperature around the children, the measurement method of wearable sensors worn by young children, as described in Section 2.2.2, was carried out. Five one-year-old children and four five-years-old children participated in the measurement in summer, and six one-year-old children and five five-years-old children participated in the measurement as observation objects in winter (Table 6). Meanwhile, the schedule, activities, and positions of observation objects children wearing wearable sensors every 10 min throughout the day were recorded (Table 7 and Figure 7). 

**Figure 8:**
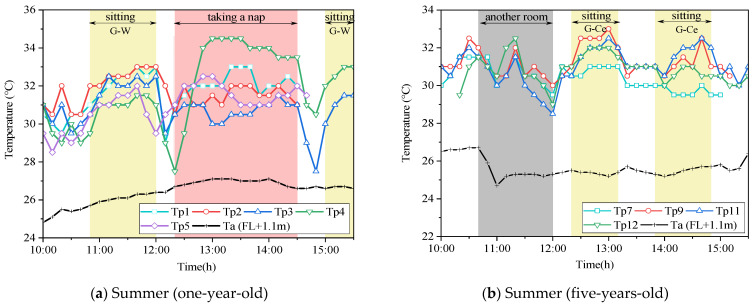
The typical day temperature in the pocket of children’s right thigh in summer. (**a**) One-year-old child; (**b**) five-years-old child. Source: Own source.

The temperature of the children’s right thigh pocket is shown in Figure 8. Colored dots and lines represent the temperature of children’s pockets measured by wearable sensors, and the number corresponds to the number of the measured child. The indoor temperature measured at 1.1 m from the ground (Ta) is represented by a black dotted line. Since the purpose was to investigate the actual temperature distribution around children in air-conditioned classrooms, we deleted some data based on observing children’s positions and behavior. The deleted data included children’s time such as in the bathrooms, outdoors, and in other classrooms. The nap time of one-year-old children was from 12:30 to 14:30. Because the children were covered by a quilt, the measured pocket temperature was significantly higher than that in other time periods. The data in this period were thus also deleted. Meanwhile, due to the change of classroom layout, the positions of the children changed greatly in summer and winter. For example, in the classroom of one-year-old children, in summer, the desks were placed on one side of the window, while the central area was used for naps. In winter, children’s desks were placed on the side near the corridor, while the area near the window was used for naps. In addition, due to the size, gender, position, and behavior of the children, the temperatures measured in the pockets of different children under a steady state were different. The maximum temperature difference was 2.5 °C in summer and 6.25 °C in winter.

**Figure 9:**
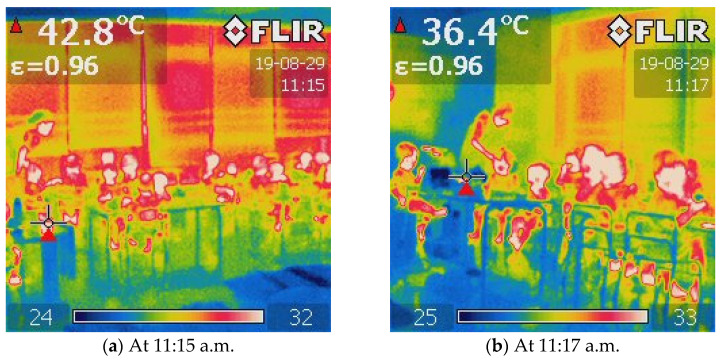
Thermography of the typical time for the one-year-old’ classroom (window side). (**a**) At 11:15 a.m.; (**b**) at 11:17 a.m. Source: The thermography was taken during measurements by authors.

Figure 8a shows the diurnal variation of the classroom pocket temperature of one-year-old children in summer. The yellow parts of 10:50–12:00 and 15:00–15:30 represent times when the children were sitting in fixed locations. The temperature measured in the pockets increased during this period. During this period, children were closer to the windows (Figure 7). The increasing temperature could be due to the solar radiation from the window side and higher outdoor temperature. This could be due to the fact that classrooms other than 1-year-old children’s classrooms have open balconies that serve as shields from solar radiation. However, the 1-year-old children’s classroom does not have an open balcony, which results in inadequate solar shading. A typical example shows the thermography of the window side in the classroom (Figure 9). The thermography illustrates that the classroom was well exposed to sunlight at this time, and the windows were visible as red or yellow overall, the floor near the windows was yellow or green, and the areas away from the windows were green or blue. It indicates a horizontal temperature distribution in the classroom, with the side near the window being hotter than the area away from the window. This has a great impact on children’s thermal environment and thermal comfort. Hence, specific attention should be paid to the thermal environment when children move near the window side.

Figure 8b shows the daily variation of classroom pocket temperature in summer for five-years-old children. The yellow parts from 12:20 to 13:10 and 13:50 to 14:50 represent five-years-old children sitting at fixed locations in the classroom. Meanwhile, the temperature of children’s pocket No. 7 (light blue line) is 2.5 °C lower than that of other children. Combined with the child location map recorded in Figure 7, we consider that this could result from child No. 7 being seated right below the air outlet of the air conditioning system. This result indicated that the surrounding environment could affect the temperature around children. In addition, sub-cooling will reduce the thermal comfort of occupants [37].

In addition, we should reconsider a suitable air conditioning system for nursery schools. The results of the actual measurements showed that most of the conventional air conditioning systems used in nursery schools have certain disadvantages, such as direct air blowing from the air conditioning vents and the vicinity of the air conditioning vents being colder than the surroundings during cooling. These disadvantages are not sufficient to create a suitable thermal environment for children. Therefore, some other practical radiation air-conditioning systems may be more worthy to be applied, such as utilizing a thermal radiation effect for air-conditioning with radiant panels mounted on the ceiling, which has the advantage of eliminating draughts [50]. Meanwhile, the advantage of creating a smaller vertical distribution in the air temperature during heating was verified in Section 3.2.1 of this study. It demonstrated that radiation air-conditioning systems can obtain more occupant comfort votes while creating a suitable thermal environment than convection air conditioning systems. Meanwhile, they have a great potential to save energy and can significantly reduce building energy consumption [50]. These advantages may be beneficial in creating a suitable thermal environment for children. Meanwhile, actual measurements showed that window-side conditions and cooling systems during a hot summer may make children feel uncomfortable. Hence, nursery teachers should prevent children from moving around on the window side and near air conditioning vents by setting up individualized approaches such as changing the classroom layout and activity areas, which may be effective in helping to improve children’s thermal comfort. On the other hand, in terms of building design, the approach of improving the thermal insulation of windows and facades of buildings and sun shading in summer could also contribute to improving the thermal environment for the children.

**Figure 10:**
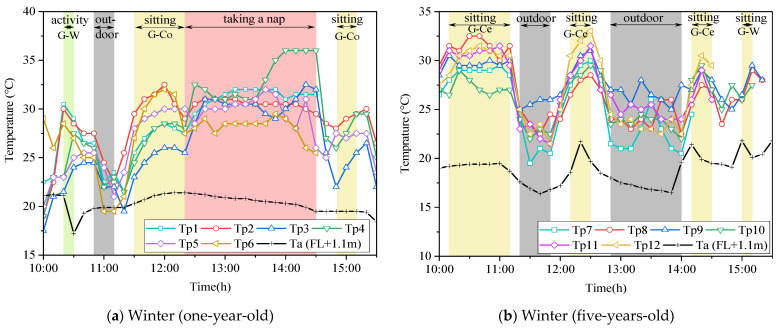
The typical day temperature in the pocket of children’s right thigh in winter. (**a**) One-year-old children; (**b**) five-years-old children. Source: Own source.

Figure 10a shows the daily variation of the temperature in the pocket of one-year-old children in the classroom in winter. The low pocket temperature after returning to the room at 11:10 could be owed to the sensors being cooled by the outside air, while the measured children did not wear cold-proof clothes during outdoor activities, thus resulting in a drop in temperature, and the measured data also showed the low values. Therefore, the 20 min after returning to the room from outside activities were regarded as an unstable state and were excluded from this data analysis. In addition, natural ventilation was conducted at 10:30, and the indoor temperature at FL + 1.1 m decreased significantly. Then, the children moved to the side of the window at 10:20. Except for child No. 1, the maximum pocket temperature of the other five children moving on one side of the corridor from 11:30 to 12:20 was higher than that at 10:20. However, since natural ventilation had not been carried out at 10:20, the indoor temperature at FL + 1.1 m was higher than at 11:30. This means that the temperature in the classroom near the corridor side is high in winter, which could be due to the heating state of the corridor.

Figure 10b shows the daily variation in temperature in the pocket of five-years-old children in the classroom in winter. The yellow parts represent times when the children were having classes or doing work at their desks. Especially between 14:10 and 15:10, when children gather in front of their desks, the temperature in their pockets rises significantly, which indicates that the pocket temperature could also be affected by the surrounding human bodies. Providing appropriate indoor thermal conditions is conducive to children’s health and learning [10,12]. Meanwhile, an uneven indoor thermal environment and children’s intensive factors can lead to children’s thermal comfort being lower than expected. Additionally, children’s autonomic thermoregulation function is not yet mature, so the action thermoregulation response plays a very important role in maintaining life [31]. In the conclusion of the questionnaire survey, we discussed that the basis of adaptive comfort behavior is mostly based on the teachers’ own thermal feeling, and the teachers do not know whether the temperature is appropriate for children. According to the measurement results, we suggest that teachers improve the thermal comfort of gathered children through thermal adaptive behavior.

## 4. Conclusions

The indoor environment has a great impact on the physical, mental, and work efficiency of occupants. Meanwhile, due to psychological and physical differences, children are more vulnerable to the influence of the surrounding environment than adults. Therefore, a nursery school in Fukuoka, Japan was selected as the research object. The actual thermal environment of children aged one to five in the classroom was evaluated based on the measured data in winter and summer. There are significant differences in physical characteristics between children and adults. Meanwhile, children lack autonomy in terms of thermal adaptive comfort behavior. Hence, to provide a suitable indoor environment for children, the evaluation of indoor thermal comfort and the cognition of children’s thermal comfort by teachers engaged in nursing work are more important. Through a questionnaire survey of teachers, this paper analyzed and compared the relationship between teachers’ thermal adaptation behavior and children’s thermal sensation. At the same time, compared with the traditional fixed-point measurement method, a method of wearable sensors for children was proposed to measure the indoor temperature distribution in summer and winter. Through the statistical analysis of the horizontal and vertical temperature in the classroom, the effective thermal adaptation behavior of teachers for the specific environment around children was clarified. The main conclusions of this study are shown as follows:

1. Based on the questionnaire survey, nursery teachers’ adaptability and comfort behaviors (such as air conditioner switch on/off, adjusting the temperature of the air conditioner, or window opening/closing) to indoor temperature were mainly judged by their own thermal feelings, which could bring a physiological burden to the children.
2. The uneven indoor vertical temperature distribution could be significantly reduced by using air conditioning and floor heating simultaneously, which may help the nursery create and maintain a comfortable thermal environment for the children.
3. Solar radiation, outdoor weather, and cold air blowing from the air conditioner outlet led to uneven indoor temperature distribution. Over-cooling or over-heating reduces the thermal comfort of occupants. Therefore, specifical attention should be paid to the thermal environment when children are close to the window and near the air outlet of the air conditioner. Therefore, nursery schools and nursery teachers should prevent children from moving around on the window side and near air conditioning vents during the hot summer by setting up individualized methods such as changing the classroom layouts and activity areas for children. In addition, a more suitable air-conditioning system for nursery schools should be reconsidered to reduce the above-mentioned drawbacks of conventional air conditioning systems, and we cited a radiation air-conditioning system as having the advantage of eliminating airflow, which can improve the current drawbacks of direct air blowing and help create a suitable thermal environment for children. On the other hand, in terms of building design, the approach of improving the thermal insulation of windows and facades of buildings and sun shading in summer could also contribute to improving the thermal environment for the children.
4. The density of occupants may cause the temperature around the human body to be relatively high. We suggest that teachers can improve the thermal comfort of gathered children through thermal adaptive behavior.

The study results can emphasize the importance of creating a suitable indoor environment strategy from the practical perspective of children’s thermal environment while providing valuable information for designers and nursery managers to formulate effective indoor thermal environment strategies from the perspective of children.

## Figures and Tables

**Table 1:** Weather in Fukuoka City during the field study.

Year	Date	Temperature (°C)			Humidity (%)			Wind Speed (m/s)	Precipitation (mm)	Sunshine Duration (h)
		Mean	Min	Max	Mean	Min	Max	Mean	Total	Total
2019	28 August	25.7	23.7	29.3	92	72	100	1.9	86.0	0.0
	29 August	25.3	24.0	29.4	91	65	100	1.8	43.0	1.6
	30 August	25.6	23.2	29.6	79	44	82	1.7	0.5	1.7
	September	25.0	23.0	29.2	90	71	100	1.5	4.5	0.8
	3 September	27.5	23.2	31.9	78	59	100	2.4	0.5	8.2
	4 September	27.2	24.5	31.2	80	61	94	2.0	0.0	4.4
	5 September	28.4	25.1	33.2	75	56	92	2.2	1.0	8.4
	6 September	29.8	25.8	34.8	63	40	84	3.2	0.0	4.3
	9 September	30.0	25.9	34.8	71	71	88	2.3	0.0	10.0
2020	18 February	4.8	2.1	8.4	57	46	68	3.6	0.0	3.0
	19 February	7.0	1.7	14.2	66	42	82	2.2	0.0	10.1
	20 February	9.6	6.0	14.8	64	44	79	2.4	0.0	9.1
	21 February	12.5	4.4	18.5	50	9	79	2.7	0.0	10.0
	25 February	15.2	11.5	19.7	63	41	100	3.1	7.0	0.9

**Table 2:** Profile of the instrument parameters.

Instrument	Parameters	Accuracy	Resolution
Thermo Recorder TR-72 nw (T&D Corporation, Matsumoto, Japan)	Air temperature	±0.5 °C	0.1 °C
Thermo Recorder TR-72 nw	Relative humidity	±5% RH	1% RH
Thermochron Type-G (KN Laboratories, Inc., Osaka, Japan)	The temperature in the pocket of the right thigh	±1 °C	0.5 °C
CHINO, CPA (CHINO Corporation, Tokyo, Japan)	Thermography camera	±2 °C	0.15 °C

**Table 3:** Questionnaire results about the nursery school.

Question	First Majority Response		Second Majority Response		Third Majority Response	
	Description 1/2pt	%	Description	%	Description	%
Responsible class	Nursery school directors	33.8	Under one year old	16.4	Five years old	15.1
What is the type of air-conditioning	Ceiling cassette	87.3	Ceiling and floor	7.0	Floor standing	4.2
Setting rate of sunshade	Facilities installed	94.4	Facilities not installed	5.6	-	-
Types of sunshades	Window curtains	67.4	Roller shutter	11.2	Window shades	6.7
Is the setting temperature of air-conditioning adaptive or fixed (summer)	Adaptive	50.7	Fixed	49.3	-	-
(winter)	Adaptive	57.7	Fixed	42.3	-	-
What are the reasons for starting or changing air-conditioning temperature (multi-choice)	The questionnaire respondents are feeling cold or hot	26.9	The children are feeling cold or hot	25.1	The indoor thermometer shows high or low temperatures	22.0
Problems in the operation of air conditioning system (multi-choice)	I do not know whether the set temperature of the air conditioner matches the temperature of the child’s space	20.9	If they use it for a long time, they worry about whether the children feel comfortable or not	17.6	The smell of air conditioning and mold	15.4

**Table 4:** Questionnaire results about the indoor thermal environment.

Question	Description	Median	Mean	SD
How do you feel the indoor air temperature in summer? (When air-conditioning is on)	1 (cold) to 7 (hot)	4	3.70	0.64
(When air-conditioning is off)	1 (cold) to 7 (hot)	2	1.75	0.91
How do you feel the indoor air temperature in winter? (When air-conditioning is on)	1 (cold) to 7 (hot)	4	4.06	0.45
(When air-conditioning is off)	1 (cold) to 7 (hot)	6	5.18	1.62
Are you satisfied with the indoor thermal environment in summer? (When air-conditioning is on)	1 (strongly dissatisfied) to 7 (strongly satisfied)	2	2.07	0.82
(When air-conditioning is off)	1 (strongly dissatisfied) to 7 (strongly satisfied)	6	4.68	1.95
Are you satisfied with the indoor thermal environment in winter? (When air-conditioning is on)	1 (strongly dissatisfied) to 7 (strongly satisfied)	2	2.16	0.98
(When air-conditioning is off)	1 (strongly dissatisfied) to 7 (strongly satisfied)	5	4.22	2.07
Do you think the indoor thermal environment is comfortable in summer? (When air-conditioning is on)	1 (no), 2 (not sure), 3 (yes)	3	2.86	0.43
(When air-conditioning is off)	1 (no), 2 (not sure), 3 (yes)	1	1.35	0.61
Do you think the indoor thermal environment is comfortable in winter? (When air-conditioning is on)	1 (no), 2 (not sure), 3 (yes)	3	2.85	0.36
(When air-conditioning is off)	1 (no), 2 (not sure), 3 (yes)	1	1.45	0.66
Have you ever noticed vertical temperature differences?	1 (never), 2 (sometimes), 3 (often), 4 (always)	3	2.68	0.86
Have you ever noticed horizontal temperature differences?	1 (never), 2 (sometimes), 3 (often), 4 (always)	3	3.24	0.69

**Table 5:** Profile of measurement and methods adopted to adjust the indoor temperature.

Season	Period	Way of Adjusting Indoor Air Temperature	
		One-Year-Old Children’s Classroom	Five-Years-Old Children’s Classroom
Summer	28–30 August, 2–6 September, and 9 September 2019	Air conditioning	Air conditioning
Winter	18 February, 20–21 February 2020, and 25 February 2020	Air conditioning	Air conditioning
	19 February 2020	Air conditioning + floor heating	Air conditioning

**Table 6:** Typical measurement object.

Season	Date	Children’s Classroom	Measurement Object (Number)
Summer	29 August 2019	One-year-old	1, 2, 3, 4, 5.
		Five-years-old	7, 9, 11, 12.
Winter	19 February 2020	One-year-old	1, 2, 3, 4, 5. 6 (only for winter measurement)
		Five-years-old	7, 9, 11, 12. 8 and 10 (only for winter measurement)

**Table 7:** Recorded schedule of typical child activities in a day.

Date	Time	Schedule	Time	Schedule
29 August	One-year-old		Five-years-old	
	9:30~	Measurement starts Gymnastics	9:30~	Measurement starts Free play
	9:50~	Having a snack	9:40~	Reading
	10:10~	Dancing	9:50~	Singing
	10:20~	Free play	10:10~	Exercise
	10:50~	Preparing for lunch	10:40~	Move to another classroom activity
	11:30~	Lunch	12:10~	Preparing for lunch
	12:10~	Free play	12:20~	Lunch
	12:20~	Taking a nap	13:10~	Tidying up
	14:30~	End the nap and free play	13:20~	Free play
	14:40~	Free play	13:50~	Reading
	14:50~	Tidying up	14:50~	Free play
	15:00~	Having a snack	15:00~	Outdoor
	15:30~	Measurement end	15:30~	Measurement end
19 February	One-year-old		Five-years-old	
	10:00~	Measurement starts Having a snack	9:30~	Measurement starts
	10:10~	Free play	9:40~	Reading
	10:20~	Reading	9:50~	Singing
	10:30~	Gymnastics	10:10~	Writing
	10:50~	Outdoor activity	11:20~	Outdoor activity
	11:10~	Free play	11:50~	Enter indoors and prepare for lunch
	11:30~	Lunch	12:10~	Lunch
	12:20~	Taking a nap	12:40~	Free play
	14:30~	End the nap and free play	12:50~	Outdoor activity
	14:40~	Free play	14:00~	Free play
	14:50~	Having a snack	14:20~	Move to another classroom activity
	15:20~	Measurement end	15:00~	Having a snack
			15:20~	Measurement end

## Data Availability

Not applicable.

## References

[1] Branco P.T., Alvim-Ferraz M.C., Martins F.G., Ferraz C., Vaz L.G., Sousa S.I. (2020). Impact of indoor air pollution in nursery and primary schools on childhood asthma. Sci. Total Environ..

[2] Huang L., Zhu Y., Ouyang Q., Cao B. (2012). A study on the effects of thermal, luminous, and acoustic environments on indoor environmental comfort in offices. Build. Environ..

[3] Annesi-Maesano I., Agabiti N., Pistelli R., Couilliot M.-F., Forastiere F. (2003). Subpopulations at increased risk of adverse health outcomes from air pollution. Eur. Respir. J..

[4] Inoue Y. (2004). Prevention of heat illness in children and the Elderly. Jpn. J. Biometeorol..

[5] Copple C. (2003). Fostering young children’s representation, planning, and reflection: A focus in three current early childhood models. J. Appl. Dev. Psychol..

[6] Korsavi S.S., Montazami A. (2020). Children’s thermal comfort and adaptive behaviours; UK primary schools during non-heating and heating seasons. Energy Build..

[7] Vásquez N.G., Rupp R.F., Díaz L.A., Cardona A.G., Arenas D.M. Testing a method to assess the thermal sensation and preference of children in kindergartens. Proceedings of the 30th International PLEA Conference: Sustainable Habitat for Developing Societies: Choosing the Way Forward—Proceedings.

[8] Nam I., Yang J., Lee D., Park E., Sohn J.-R. (2015). A study on the thermal comfort and clothing insulation characteristics of preschool children in Korea. Build. Environ..

[9] Madureira J., Paciência I., Rufo J., Ramos E., Barros H., Teixeira J.P., de Oliveira Fernandes E. (2015). Indoor air quality in schools and its relationship with children’s respiratory symptoms. Atmos. Environ..

[10] Fabbri K. (2015). Indoor Thermal Comfort Perception. A Questionnaire Approach Focusing on Children.

[11] Yun H., Nam I., Kim J., Yang J., Lee K., Sohn J. (2014). A field study of thermal comfort for kindergarten children in Korea: An assessment of existing models and preferences of children. Build. Environ..

[12] Fabbri K. (2013). Thermal comfort evaluation in kindergarten: PMV and PPD measurement through datalogger and questionnaire. Build. Environ..

[13] Liang H.-H., Lin T.-P., Hwang R.-L. (2012). Linking occupants’ thermal perception and building thermal performance in naturally ventilated school buildings. Appl. Energy.

[14] Huang K.-T., Huang W.-P., Lin T.-P., Hwang R.-L. (2015). Implementation of green building specification credits for better thermal conditions in naturally ventilated school buildings. Build. Environ..

[15] Al-Rashidi K., Loveday D., Al-Mutawa N. (2012). Impact of ventilation modes on carbon dioxide concentration levels in Kuwait classrooms. Energy Build..

[16] Fong M., Hanby V., Greenough R., Lin Z., Cheng Y. (2015). Acceptance of thermal conditions and energy use of three ventilation strategies with six exhaust configurations for the classroom. Build. Environ..

[17] Frontczak M., Wargocki P. (2011). Literature survey on how different factors influence human comfort in indoor environments. Build. Environ..

[18] Zomorodian Z.S., Tahsildoost M., Hafezi M. (2016). Thermal comfort in educational buildings: A review article. Renew. Sustain. Energy Rev..

[19] Teli D., Jentsch M.F., James P.A. (2014). The role of a building’s thermal properties on pupils’ thermal comfort in junior school classrooms as determined in field studies. Build. Environ..

[20] Li X., Chen Q. (2021). Development of a novel method to detect clothing level and facial skin temperature for controlling HVAC systems. Energy Build..

[21] Li W., Zhang J., Zhao T., Liang R. (2018). Experimental research of online monitoring and evaluation method of human thermal sensation in different active states based on wristband device. Energy Build..

[22] Cosma A.C., Simha R. (2018). Thermal comfort modeling in transient conditions using real-time local body temperature extraction with a thermographic camera. Build. Environ..

[23] Cosma A.C., Simha R. (2018). Machine learning method for real-time non-invasive prediction of individual thermal preference in transient conditions. Build. Environ..

[24] Wang F., Zhu B., Li R., Han D., Sun Z., Moon S., Gong Z., Yu W. Smart control of indoor thermal environment based on online learned thermal comfort model using infrared thermal imaging. Proceedings of the 2017 13th IEEE Conference on Automation Science and Engineering (CASE).

[25] Cheng X., Yang B., Hedman A., Olofsson T., Li H., Van Gool L. (2019). NIDL: A pilot study of contactless measurement of skin temperature for intelligent building. Energy Build..

[26] Sugimoto C. Human sensing using wearable wireless sensors for smart environments. Proceedings of the International Conference on Sensing Technology, ICST.

[27] Sim S.Y., Koh M.J., Joo K.M., Noh S., Park S., Kim Y.H., Park K.S. (2016). Estimation of Thermal Sensation Based on Wrist Skin Temperatures. Sensors.

[28] Smith C.J. (2019). Pediatric Thermoregulation: Considerations in the Face of Global Climate Change. Nutrients.

[29] Berko J., Ingram D.D., Saha S., Parker J.D. (2014). Deaths attributed to heat, cold, and other weather events in the United States, 2006–2010. Natl. Health Stat. Rep..

[30] Falk B. (1998). Effects of Thermal Stress During Rest and Exercise in the Paediatric Population. Sports Med..

[31] Fukazawa T., Ikeda S., Kim S., Tochihara Y. (2009). Seasonal clothing variation and thermal resistance of clothing ensembles of infants living in Kyushu. J. Home Econ. Jpn..

[32] Teli D., James P.A., Jentsch M.F. (2013). Thermal comfort in naturally ventilated primary school classrooms. Build. Res. Inf..

[33] Aparicio-Ruiz P., Barbadilla-Martín E., Martín J.G., Sanz J.M. (2021). A field study on adaptive thermal comfort in Spanish primary classrooms during summer season. Build. Environ..

[34] Kirin Nursery School, SKT Co., Ltd., West Japan Telecom and Telephone Corporation, Kyushu Institute of Technology (2018). Fukuoka City LoRaWANTM Was Used to Realize the Trial Operation of IoT Nursery School. https://www.ntt-west.co.jp/newscms/fukuoka/7221/20180228.pdf.

[35] (2018). L. and W. (Japan) Ministry of Health, Guidelines for Infectious Disease Control in Nurseries, Ministry of Health, Labour and Welfare (Japan). https://www.mhlw.go.jp/file/06-Seisakujouhou-11900000-Koyoukintoujidoukateikyoku/0000201596.pdf.

[36] (2008). L. and W. (Japan) Ministry of Health, Building Environmental Health Management Standard, Ministry of Health, Labour and Welfare (Japan). https://www.mhlw.go.jp/bunya/kenkou/seikatsu-eisei10/index.html.

[37] Ole Fanger P. (1970). Thermal comfort. Analysis and Applications in Environmental Engineering.

[38] Alsmo T., Alsmo C. (2014). Ventilation and Relative Humidity in Swedish Buildings. J. Environ. Prot..

[39] Kalamees T., Vinha J., Kurnitski J. (2006). Indoor Humidity Loads and Moisture Production in Lightweight Timber-frame Detached Houses. J. Build. Phys..

[40] Bornehag C.-G., Blomquist G., Gyntelberg F., Järvholm B., Malmberg P., Nordvall L., Nielsen A., Pershagen G., Sundell J. (2001). Dampness in Buildings and Health.Nordic interdisciplinary review of the scientific evidence on associations between exposure to “dampness” in buildings and health effects (NORDDAMP). Indoor Air.

[41] Bornehag C.-G., Sundell J., Bonini S., Custovic A., Malmberg P., Skerfving S., Sigsgaard T., Verhoeff A. (2004). Dampness in buildings as a risk factor for health effects, EUROEXPO: A multidisciplinary review of the literature (1998–2000) on dampness and mite exposure in buildings and health effects. Indoor Air.

[42] Derby M.M., Hamehkasi M., Eckels S., Hwang G.M., Jones B., Maghirang R., Shulan D. (2016). Update of the scientific evidence for specifying lower limit relative humidity levels for comfort, health, and indoor environmental quality in occupied spaces (RP-1630). Sci. Technol. Built Environ..

[43] Arundel A.V., Sterling E.M., Biggin J.H., Sterling T.D. (1986). Indirect health effects of relative humidity in indoor environments. Environ. Health Perspect..

[44] Wolkoff P. (2018). Indoor air humidity, air quality, and health—An overview. Int. J. Hyg. Environ. Health.

[45] Andersen I., Msc G.R.L., Jensen P.L., Proctor D.F. (1974). Human Response to 78-Hour Exposure to Dry Air. Arch. Environ. Health Int. J..

[46] Abusharha A.A., Pearce E.I. (2013). The Effect of Low Humidity on the Human Tear Film. Cornea.

[47] Liu C., Zhang Y., Sun L., Gao W., Jing X., Ye W. (2021). Influence of indoor air temperature and relative humidity on learning performance of undergraduates. Case Stud. Therm. Eng..

[48] (2019). C.S.S. and T. (Japan) Ministry of Education, Statistical Investigation Report on School Health Care, Ministry of Education, Culture, Sports, Science and Technology (Japan). https://www.mext.go.jp/content/20210728-mxt_chousa01-000013187_1.pdf.

[49] (2005). Ergonomics of the Thermal Environment Analytical Determination and Interpretation of Thermal Comfort Using Calculation of the PMV and PPD Indices and Local Thermal Comfort Criteria.

[50] Imanari T., Omori T., Bogaki K. (1999). Thermal comfort and energy consumption of the radiant ceiling panel system. Energy Build..

